# Supplementary material for: Quantum sensing and metrology with free electrons
Source: Nat Commun. 2025 Dec 23;17:868. doi: 10.1038/s41467-025-67585-9 (PMC12828002; doi:10.1038/s41467-025-67585-9)
Supplement: Supplementary file 1 — Supplementary Information [file 41467_2025_67585_MOESM1_ESM.pdf]

Quantum Sensing and Metrology with Free Electrons

– SUPPLEMENTARY INFORMATION –

Cruz I. Velasco[1] and F. Javier García de Abajo[1,2,*]

[1]ICFO-Institut de Ciencies Fotoniques, The Barcelona Institute of Science and Technology, 08860 Castelldefels (Barcelona), Spain

[2]ICREA-Institució Catalana de Recerca i Estudis Avançats, Passeig Lluís Companys 23, 08010 Barcelona, Spain

Contents

S1. Electron transmission probabilities in QUAFE 1
A. Electron–waveguide interaction 1
B. Electron beam splitting/mixing 2
C. Electron current in the configuration of Fig. 4a 3
D. Electron current in the configuration of Fig. 4b 3
E. The effect of photon loss in QUAFE measurements 4

S2. Electron trajectory 4

S3. Image force 5

S4. Effective interaction length 6

References 7

SUPPLEMENTARY FIGURES 8

S1. ELECTRON TRANSMISSION PROBABILITIES IN QUAFE

We consider a relativistic incident electron of kinetic energy and momentum tightly centered around the values $\mathcal{E}_0 = m_e c^2(\gamma - 1)$ and $\hbar \mathbf{q}_0 = m\mathbf{v}\gamma$, respectively, where $\mathbf{v}$ is the electron velocity, $\gamma = 1/\sqrt{1-(v/c)^2}$ is the Lorentz factor, c is the speed of light, and m_e is the electron mass. In what follows, we assume paraxial propagation and adopt the nonrecoil approximation, such that the electron velocity v along the electron-beam (e-beam) axis z can be regarded as constant, while transverse velocity components are small compared with v. We combine two central elements in our work: the aloof interaction with an optical waveguide and the lateral splitting/mixing of e-beams into different diffraction directions. In this section, we first describe each of these elements (Sec. S1 A and S1 B) and then apply them to calculate the QUAFE electron current under the configurations of Fig. 4 in the main text (Sec. S1 C and S1 D), including the effect of inelastic light absorption in the waveguide (Sec. S1 E).

A. Electron–waveguide interaction

The electromagnetic modes supported by the waveguide are characterized by photon energies $\hbar\omega_n$ and electric-field distributions $\mathbf{E}_n(\mathbf{r})$. Although each band n consists of a continuum of modes as a function of parallel wave vector, the electron can only generate modes with wave vectors around a central value determined by the phase-matching condition $k_\parallel = \omega_n/v$ for each band n [1]. Actually, because the electron interacts within a limited time with the waveguide (see Sec. S2), it generates a finite distribution of wave vectors and frequencies around these values (i.e., $\Delta\omega_n \sim v/L_n^{\rm eff}$ for a mode-dependent interaction length $L_n^{\rm eff}$;[1] see Sec. S4). These finite distributions of wave vectors define the electron-generated photon modes (one per band n). In addition, the generation of a photon in mode n subtracts an energy $\hbar\omega_n$ from the incident electron energy $\mathcal{E}_0$. Therefore,

*Electronic address: javier.garciadeabajo@nanophotonics.es

[1] Under the conditions of Fig. 1 in the main text, considering 200 keV electrons and assuming an interaction length $L_0^{\rm eff} = 1$ mm in the $n = 0$ mode (photon energy $\hbar\omega_0 \approx 0.5$ eV), we have $\Delta\omega_0 \approx 0.1$ meV$/\hbar \ll \omega_0$.

the electron–waveguide system can be described in terms of photon number states $|\{N_n\}\rangle$ associated with the photon populations N_n in different waveguide modes n. Each of these states is entangled with an electron energy $\mathcal{E}_0 - \sum_n N_n \hbar\omega_n$. In addition, we assume that the interaction does not affect the lateral electron wave function, so we do not need to consider the electron state explicitly (i.e., each ket $|\{N_n\}\rangle$ already determines the electron state).

From these considerations, we can write the interaction Hamiltonian as

$$\hat{\mathcal{H}}_{\rm int}(\mathbf{r}) = -{\rm i}e \sum_n \omega_n^{-1}\,\mathbf{v}\cdot\Big[\mathbf{E}_n(\mathbf{r})\hat{a}_n - \mathbf{E}_n^*(\mathbf{r})\hat{a}_n^\dagger\Big]$$

in terms of photon creation and annihilation operators $\hat{a}_n^\dagger$ and $\hat{a}_n$ for each mode n, understanding that the action of these operators also affects the electron energy. Following the methods of ref. [2] and starting from an electron with energy $\mathcal{E}_0$ and the waveguide in the vacuum state (i.e., $|\psi(t\to-\infty)\rangle = |0\rangle$), the post-interaction state is given by [2]

$$|\psi(t\to\infty)\rangle = \left[\prod_n {\rm e}^{{\rm i}\chi_n}\hat{\mathcal{S}}_n^{\rm int}(\beta_n)\right]|\psi(t\to-\infty)\rangle \tag{S1a}$$

[Eq. (2) in the main text], where χ_n is a global phase that can be absorbed in the electron components, and

$$\hat{\mathcal{S}}_n^{\rm int}(\beta_n) = {\rm e}^{-\beta_n\hat{a}_n + \beta_n^*\hat{a}_n^\dagger}$$

[Eq. (3)] is a displacement operator for mode n corresponding to a coupling coefficient

$$\beta_n = \frac{e}{\hbar\omega_n}\int_{-\infty}^{\infty} dz\;\hat{\mathbf{z}}\cdot\mathbf{E}_n(\mathbf{r}){\rm e}^{-{\rm i}\omega_n z/v} \tag{S1b}$$

[Eq. (1)]. Starting from $|0\rangle$, the interaction produces a Poissonian distribution of photon number states in each mode n, as given by [2]

$$\hat{\mathcal{S}}_n^{\rm int}(\beta_n)\,|0\rangle = {\rm e}^{-|\beta_n|^2/2}\sum_{N_n=0}^{\infty}\frac{(\beta_n^*)^{N_n}}{\sqrt{N_n!}}\,|N_n\rangle\,, \tag{S1c}$$

[Eq. (4)], which corresponds to an average final population $\langle N_n\rangle = |\beta_n|^2$. In addition, multiple interations can be straightforwardly incorporated using the result

$$\hat{\mathcal{S}}_n^{\rm int}(\beta_n')\hat{\mathcal{S}}_n^{\rm int}(\beta_n) = {\rm e}^{{\rm i}{\rm Im}\{\beta_n'\beta_n^*\}}\hat{\mathcal{S}}_n^{\rm int}(\beta_n'+\beta_n), \tag{S2}$$

which can be derived using the Baker-Campbell-Hausdorff identity.

In Eq. (S1b), the field is evaluated at a transverse electron position (x,y) that varies with the distance z traveled along the waveguide direction. Now, as we show below (Secs. S1 C-S1 E), the QUAFE electron transmission probabilities only depend on the average number of created photons per mode $\langle N_n\rangle$ and not on the phase of β_n (assuming the same interaction parameters, and therefore the same coefficients β_n, at points 1 and 2 in Fig. 4). Therefore, rather than calculating β_n from Eq. (S1b), we use a more direct method to compute $\langle N_n\rangle$ by integrating the photon-generation probability per unit path length over the electron trajectory (Sec. S4).

B. Electron beam splitting/mixing

We introduce an e-beam splitter and a mixer in each of the two configurations considered in Fig. 4. These elements need to be coordinated: the two e-beam paths produced by the splitter (A and B) must arrive at the mixer along directions that can be transmitted along a common central path (C). For splitters and mixers based on transmission gratings, this coordination can be achieved by properly adjusting the angles of incidence and the grating periods, and additionally, with a proper design of the gratings, the splitter produces primarily only two paths [3–5].

The splitter prepares the incident electron as a superposition of two paths, so the state of the system becomes $(|\mathcal{E}_0, A\rangle + |\mathcal{E}_0, B\rangle)\otimes|0\rangle$, where the rightmost ket refers to the waveguide(s) initial vacuum state, the longitudinal electron state is associated with a kinetic energy $\mathcal{E}_0$, which is the same in both paths, and the transverse wave function corresponds to the split paths A and B. After interaction with the waveguide(s), the system evolves into a state $\sum_j\big(a_j\,|\mathcal{E}_0 - E_j, A\rangle + b_j\,|\mathcal{E}_0 - E_j, B\rangle\big)\otimes|j\rangle$, where each photonic state $|j\rangle$ (in one or two waveguides, depending on the configuration) contains photons totaling an energy E_j that is subtracted from the electron energy, a_j and b_j are expansion coefficients, and we neglect the variation in transverse wave function produced by electron–photon exchanges. After mixing, both paths A and B are merged into a single path C with a transmission coefficient t_C (assumed to be identical for both paths and nearly constant over the electron energy range under consideration). By disregarding all other diffraction paths produced by the mixer (i.e., we post-select path C by only recording electrons emerging with the corresponding transmission angle), the mixed electron state becomes $\sum_j t_C\,(a_j + b_j)\,|\mathcal{E}_0 - E_j, C\rangle\otimes|j\rangle$.

C. Electron current in the configuration of Fig. 4a

This configuration involves an e-beam split into two paths A and B, each of them interacting with the same waveguide at two consecutive points 1 and 2 at times t_1 and t_2, respectively, supplemented by a photonic phase shift at an intermediate time t_i. The waveguide is initially unpopulated, so the state before the first interaction (after the electron passes through the first beam splitter) is

$$|\psi(t<t_1)\rangle = \frac{1}{\sqrt{2}}\big(\,|A\rangle + |B\rangle\,\big)\otimes|0\rangle\,,$$

where $|A\rangle$ and $|B\rangle$ refer to the transverse components of the electron wave function, assumed to be unchanged by the interaction with the waveguide.

At the first interaction point 1, path A approaches the waveguide and couples to its guided modes according to Eqs. (S1), while path B remains far from the waveguide, so the state becomes

$$|\psi(t_1<t<t_i)\rangle = \frac{1}{\sqrt{2}}\Big[\,|A\rangle\otimes\prod_n \hat{\mathcal{S}}_n^{\rm int}(\beta_n)\,|0\rangle + |B\rangle\otimes|0\rangle\,\Big],$$

where the second line is obtained by employing Eq. (S1c).

Between the two interactions, photons propagating along the waveguide pass through a phase shifter (e.g., an impurity that we might want to analyze) that introduces a phase $\varphi_{\ell n}$ in each mode n according to the transformation $|N_n\rangle \rightarrow {\rm e}^{{\rm i}N_n\varphi_{\ell n}}\,|N_n\rangle$. Additionally, a phase φ_e is introduced in path A relative to B due, for example, to an unintended difference in electron path length or also to transmission through an actively controlled potential phase plate. The state immediately before the second interaction thus reads

$$|\psi(t_i<t<t_2)\rangle = \frac{1}{\sqrt{2}}\Big[{\rm e}^{{\rm i}\varphi_e}\,|A\rangle\otimes\prod_n \hat{\mathcal{S}}_n^{\rm int}\big(\beta_n{\rm e}^{-{\rm i}\varphi_{\ell n}}\big)\,|0\rangle + |B\rangle\otimes|0\rangle\,\Big],$$

where the action of φ_e consists in multiplying $|A\rangle$ by ${\rm e}^{{\rm i}\varphi_e}$, while the photonic phase factors, which multiply each photon-number state, are incorporated into the arguments of the interaction operators.

During the second interaction 2, path B approaches the waveguide, while path A remains far away. We assume identical coupling conditions for both interactions, such that the coupling coefficients β_n are the same. Therefore, the state produced after interaction 2 becomes

$$\begin{aligned}|\psi(t>t_2)\rangle &= \frac{1}{\sqrt{2}}\Big[{\rm e}^{{\rm i}\varphi_e}\,|A\rangle\otimes\prod_n \hat{\mathcal{S}}_n^{\rm int}\big(\beta_n{\rm e}^{-{\rm i}\varphi_{\ell n}}\big)\,|0\rangle + |B\rangle\otimes\prod_n \hat{\mathcal{S}}_n^{\rm int}\big(\beta_n\big)\,|0\rangle\,\Big] \\ &= \frac{1}{\sqrt{2}}\Big[\prod_n {\rm e}^{-\sum_n|\beta_n|^2/2}\sum_{N_n=0}^{\infty}\frac{(\beta_n^*)^{N_n}}{\sqrt{N_n!}}\Big]\Big[{\rm e}^{{\rm i}\varphi_e+{\rm i}\sum_n N_n\varphi_{\ell n}}\,|A\rangle + |B\rangle\,\Big]\otimes|\{N_n\}\rangle\,,\end{aligned}$$

where the second line is obtained from the first one by applying Eq. (S1c).

After the two interactions, the electron paths are recombined into a single path C (see Sec. S1 B), giving rise to a final state

$$|\psi(t\rightarrow\infty)\rangle = \frac{t_C}{\sqrt{2}}\Big[\prod_n {\rm e}^{-\sum_n|\beta_n|^2/2}\sum_{N_n=0}^{\infty}\frac{(\beta_n^*)^{N_n}}{\sqrt{N_n!}}\Big]\Big[1+{\rm e}^{{\rm i}\varphi_e+{\rm i}\sum_n N_n\varphi_{\ell n}}\Big]\;|C\rangle\otimes|\{N_n\}\rangle+\cdots\,, \tag{S3}$$

where t_C is the transmission coefficient from path A and B to path C at the electron mixer, and the dots refer to components associated with other transmission paths different from C.

The electron current measured at the detector is obtained by projecting Eq. (S3) onto path C. Taking the squared absolute value of the resulting amplitude and multiplying by the incident current (I_0 after the beam splitter), this yields a measured electron current

$$I_e = I_0|t_C|^2\Big[1+\exp\Big\{-\sum_n\langle N_n\rangle\big(1-\cos\varphi_{\ell n}\big)\Big\}\cos\Big(\varphi_e+\sum_n\langle N_n\rangle\sin\varphi_{\ell n}\Big)\Big] \tag{S4}$$

[Eq. (5)], where we have replaced $|\beta_n|^2$ by $\langle N_n\rangle$.

D. Electron current in the configuration of Fig. 4b

We now consider a configuration in which the electron is again split into two paths A and B, but only path A interacts with the waveguide, and it does so twice (at points 1 and 2), while path B stays always away from

the waveguide. Between the two interaction events, phase shifts φ_e and $\varphi'_{\ell n}$ are applied to path A (relative to path B) and to each photonic mode n, respectively. Note that we introduce a prime in the optical phase to distinguish it from the one in Sec. S1 C. Proceeding in a way analogous to that section, the state of the system after the second interaction (but before the mixer) becomes

$$\begin{aligned}|\psi(t>t_2)\rangle &= \frac{1}{\sqrt{2}}\Big[\mathrm{e}^{\mathrm{i}\varphi_e}\prod_n \hat{\mathcal{S}}_n^{\rm int}(\beta_n)\hat{\mathcal{S}}^{\rm int}(\beta_n\mathrm{e}^{-\mathrm{i}\varphi'_{\ell n}})\,|A\rangle+|B\rangle\Big]\otimes|0\rangle\\ &= \frac{1}{\sqrt{2}}\Big\{\mathrm{e}^{\mathrm{i}\varphi_e}\prod_n \mathrm{e}^{\mathrm{i}|\beta_n|^2\sin\varphi'_{\ell n}}\hat{\mathcal{S}}_n^{\rm int}\big[\beta_n(1+\mathrm{e}^{-\mathrm{i}\varphi'_{\ell n}})\big]\,|A\rangle+|B\rangle\Big\}\otimes|0\rangle\,,\end{aligned}$$

where the first line incorporates the two interactions and the phase shifts in path A, while the second line is obtained by applying Eq. (S2). After the mixer, the state evolves into

$$|\psi(t\to\infty)\rangle = \frac{t_C}{\sqrt{2}}\,|C\rangle\otimes\Big\{\mathrm{e}^{\mathrm{i}\varphi_e}\prod_n\Big[\mathrm{e}^{-|\beta_n|^2\left(1+\mathrm{e}^{-\mathrm{i}\varphi'_{\ell n}}\right)}\sum_{N_n=0}^{\infty}\left(1+\mathrm{e}^{\mathrm{i}\varphi'_{\ell n}}\right)^{N_n}\frac{(\beta_n^*)^{N_n}}{\sqrt{N_n!}}\,|N_n\rangle\Big]+|0\rangle\Big\}+\cdots\,,$$

where we have applied Eq. (S1c). Finally, post-selecting path C and multiplying by I_0, we find the measured electron current

$$I_e = I_0|t_C|^2\Big[1+\mathrm{e}^{-\sum_n\langle N_n\rangle(1+\cos\varphi'_{\ell n})}\cos\Big(\varphi_e-\sum_n\langle N_n\rangle\sin\varphi'_{\ell n}\Big)\Big].$$

As stated in the main text, this expression becomes identical to Eq. (S4) if $\varphi'_{\ell n}=\pi-\varphi_{\ell n}$.

E. The effect of photon loss in QUAFE measurements

Inelastic optical losses are particularly detrimental in all-quantum-optics schemes. However, we can demonstrate that QUAFE is relatively tolerant to a certain amount of loss in the propagation of photons from interaction point 1 to 2. To see this, we introduce a probability $P_{\rm abs}$ that a given photon is inelastically lost during such a propagation. The loss probability can be incorporated by multiplying each photonic number state $|N_n\rangle$ by a factor $(1-P_{\rm abs})^{N_n/2}$ (after interaction 1 and before interaction 2). Considering the configuration of Fig. 4a in the main text and proceeding in a similar way as in Sec. S1 C, the new state of the system before recombination into path C can be written as

$$|\psi(t>t_2)\rangle = \frac{1}{\sqrt{2}}\Bigg[\prod_n \mathrm{e}^{-\sum_n|\beta_n|^2/2}\sum_{N_n=0}^{\infty}\frac{(\beta_n^*)^{N_n}}{\sqrt{N_n!}}\Bigg]\Big[(1-P_{\rm abs})^{N_n/2}\mathrm{e}^{\mathrm{i}\varphi_e+\mathrm{i}\sum_n N_n\varphi_{\ell n}}\,|A\rangle+|B\rangle\Big]\otimes|\{N_n\}\rangle+\cdots\,,$$

where the omitted terms are those that do not interfere because they are associated with the creation and subsequent inelastic loss of photons. After transmission through the mixer, the resulting current intensity becomes

$$I_e = I_0|t_C|^2\Bigg[\frac{1+\mathrm{e}^{-P_{\rm abs}\sum_n\langle N_n\rangle}}{2}+\mathrm{e}^{-\sum_n\langle N_n\rangle(1-\sqrt{1-P_{\rm abs}}\cos\varphi_{\ell n})}\cos\Big(\varphi_e+\sqrt{1-P_{\rm abs}}\sum_n\langle N_n\rangle\sin\varphi_{\ell n}\Big)\Bigg].$$

Therefore, photon losses reduce phase sensitivity of the electron current due to the factors $\sqrt{1-P_{\rm abs}}$ that multiply the sine and cosine functions in this expression. To quantify this effect, Fig. S6 shows the normalized electron current for different values of $P_{\rm abs}$.

S2. ELECTRON TRAJECTORY

We are interested in calculating the dynamics of the electron along the direction x normal to the waveguide under the conditions of Fig. 1a in the main text, assuming that the velocity v remains constant along the parallel direction z (nonrecoil approximation). From the equation of motion $d\mathbf{p}/dt=\mathbf{F}$ [6], where $\mathbf{p}=m_{\rm e}\mathbf{v}\gamma$ is the relativistic momentum, $\mathbf{v}$ is the electron velocity vector, γ is the Lorentz factor, and $\mathbf{F}$ is the force acting on the electron, we project on x and write

$$m_{\rm e}\gamma\frac{dv_x}{dt}\approx eE_{\rm DC}+F_{\rm im}(x), \tag{S5}$$

where $E_{\rm DC} > 0$ is the amplitude of the uniform repulsive DC electric field (directed toward the waveguide surface), $F_{\rm im}(x) < 0$ is the attractive image force (see details in Sec. S3), and we have used the fact that $|v_x| \ll v$ and the total electron energy loss during the interaction with the waveguide is small compared with $m_{\rm e}c^2$.

We consider an electron trajectory as shown in Fig. S2, in which the electron slowly approaches the waveguide surface ($x=0$) and is reflected at a distance $x=b$. Because the image and DC-field forces are conservative, the trajectory is symmetric along z with respect to the turning point. The x-dependent normal velocity $v_x(x) = dx/dt$ is then obtained by multiplying Eq. (S5) by dx, yielding an equation for $d(v_x^2)/dt$ that leads to

$$v_x^2(x) = \frac{2}{m_{\rm e}\gamma}\int_b^x dx' \,\big[eE_{\rm DC} + F_{\rm im}(x')\big]$$

after integration. Note that this result applies to $x \geq b$ (i.e., above the turning point). To a good approximation, we find $F_{\rm im}(x) = F_{\rm im}(b)\,(b/x)^2$ (i.e., an overall $1/x^2$ dependence of the image force, as shown in Sec. S3 and Fig. S7a), and thus, the normal velocity satisfies the closed-form relation

$$v_x^2(x) = \frac{2}{m_{\rm e}\gamma}\big[eE_{\rm DC}\,(x-b) + F_{\rm im}(b)\,b(1-b/x)\,\big], \tag{S6}$$

which guarantees the conservation of normal kinetic energy. In the absence of an image interaction, using the transformation $v_x = dx/dt \to (dx/dz)v$ and integrating over x, we find the parabolic trajectory $x_e(z) = b + eE_{\rm DC}z^2/(2m_{\rm e}\gamma v^2)$. In contrast, for finite $F_{\rm im}$, $x_e(z)$ can be explicitly written in terms of elliptic integrals. However, we do not need to use $x_e(z)$ explicitly in our calculations (see below).

S3. IMAGE FORCE

We calculate the image force acting on the electron from the lateral component of the self-induced Lorentz force $\mathbf{F}_{\rm self}(t)$ produced by the induced electromagnetic fields acting back on the electron. In a general scenario, taking the electron velocity $\mathbf{v} = v\hat{\mathbf{z}}$ along z, we have $\mathbf{F}_{\rm self}(t) = -e\big[\mathbf{E}^{\rm ind}(\mathbf{r}_e(t),t) + (\mathbf{v}/c)\times\mathbf{B}^{\rm ind}(\mathbf{r}_e(t),t)\big]$, where the induced electric and magnetic fields are evaluated at the electron position, described by a trajectory $\mathbf{r}_e(t) = \mathbf{R}_0 + \mathbf{v}t$ with $\mathbf{R}_0 = (x_0,y_0)$ denoting the e-beam coordinates in the transverse plane. Working in the frequency domain [i.e., $\mathbf{E}(\mathbf{r},\omega) = \int dt\, {\rm e}^{{\rm i}\omega t}\mathbf{E}(\mathbf{r},t)$], we write the self-consistent electric field produced by the electron as [7]

$$\mathbf{E}(\mathbf{r},\omega) = -4\pi{\rm i}\omega\int d^3\mathbf{r}'\, G(\mathbf{r},\mathbf{r}',\omega)\cdot\mathbf{j}(\mathbf{r}',\omega)$$

in terms of the electromagnetic Green tensor $G(\mathbf{r},\mathbf{r}',\omega)$ defined by

$$\nabla\times\nabla\times G(\mathbf{r},\mathbf{r}',\omega) - k^2\epsilon(\mathbf{r},\omega)G(\mathbf{r},\mathbf{r}',\omega) = -(1/c^2)\delta(\mathbf{r}-\mathbf{r}'),$$

where $k=\omega/c$ is the light wavenumber and $\epsilon(\mathbf{r},\omega)$ is the frequency- and position-dependent permittivity describing the material structure with which the electron interacts. Also, $\mathbf{j}(\mathbf{r},\omega) = -e\,\hat{\mathbf{z}}\,{\rm e}^{{\rm i}\omega z/v}\delta(x-x_0)\delta(y-y_0)$ is the electron current density in frequency space.

We are interested in the self-induced impulse experienced by the electron, which, using the expressions given above, can be written as

$$\int dt\,\mathbf{F}_{\rm self}(t) = -\frac{2e^2}{v}\int dz\int dz'\int {\rm i}\omega\, d\omega\, {\rm e}^{{\rm i}\omega(z'-z)/v}\,\Big[\mathcal{I} - \frac{{\rm i}v}{\omega}\,\hat{\mathbf{z}}\times\nabla\times\Big]\,G^{\rm ind}(\mathbf{R}_0,z,\mathbf{R}_0,z',\omega)\cdot\hat{\mathbf{z}},$$

where $\mathcal{I}$ is the 3×3 unit matrix and $G^{\rm ind}$ is the induced part of the Green tensor, obtained from G by subtracting the free-space Green tensor $-(k^2\mathcal{I}+\nabla\otimes\nabla)\,{\rm e}^{{\rm i}k|\mathbf{r}-\mathbf{r}'|}/(4\pi\omega^2|\mathbf{r}-\mathbf{r}'|)$. To derive this result, we have changed the integration variable from t to $z=vt$, used Faraday's law to write the magnetic field as $\mathbf{B}(\mathbf{r},\omega) = (-{\rm i}c/\omega)\nabla\times\mathbf{E}(\mathbf{r},\omega)$ (noticing that the permeability is unity in vacuum within the Gaussian units used in this work), and expressed the electric field in terms of the Green tensor and the electron current density. Now, rewriting $\hat{\mathbf{z}}\times\nabla\times G = \nabla(\hat{\mathbf{z}}\cdot G) - \partial_z G$ and performing the z integral by parts to transform $\partial_z \to {\rm i}\omega/v$, we obtain

$$\begin{aligned}\int dt\,\mathbf{F}_{\rm self}(t) &= -2e^2\int dz\int dz'\int_{-\infty}^{\infty} d\omega\, {\rm e}^{{\rm i}\omega(z'-z)/v}\,\nabla G_{zz}^{\rm ind}(\mathbf{R}_0,z,\mathbf{R}_0,z',\omega)\\ &= -4e^2\int dz\int dz'\int_0^{\infty} d\omega\, {\rm Re}\big\{{\rm e}^{{\rm i}\omega(z'-z)/v}\,\nabla G_{zz}^{\rm ind}(\mathbf{R}_0,z,\mathbf{R}_0,z',\omega)\big\},\end{aligned} \tag{S7}$$

where we have defined $G_{zz} = \hat{\mathbf{z}}\cdot G\cdot\hat{\mathbf{z}}$ and applied causality $[G(\mathbf{r},\mathbf{r}',-\omega) = G^*(\mathbf{r},\mathbf{r}',\omega)]$.

Reassuringly, the total energy loss (obtained in the nonrecoil approximation by multiplying the impulse by $\mathbf{v}$ and integrating over z by parts again) coincides with $-\int_0^\infty d\omega\,\hbar\omega\,\Gamma_{\rm EELS}(\mathbf{R}_0,\omega)$, where $\Gamma_{\rm EELS}(\mathbf{R}_0,\omega)=(4e^2/\hbar)\int dz\int dz'\,\cos[\omega(z-z')/v]\,{\rm Im}\big\{-G_{zz}(\mathbf{R}_0,z,\mathbf{R}_0,z',\omega)\big\}$ is the electron energy-loss probability [7] and we have used reciprocity $[G(\mathbf{r},\mathbf{r}',\omega)=G^T(\mathbf{r}',\mathbf{r},\omega)]$. The self-induced force along the velocity direction is thus responsible for electron friction. Instead, in this section, we are interested in the lateral components, which we refer to as image force.

For systems with translational invariance along $\mathbf{v}$ (e.g., our waveguide), the Green tensor depends on z and z' only via $z-z'$, so the image force is constant and can be found by dividing Eq. (S7) by the total interaction time $(1/v)\int dz$. Then, the image force reduces to

$$\mathbf{F}_{\rm im}(\mathbf{R}_0)=-4e^2v\int dz\int_0^\infty d\omega\;{\rm Re}\big\{{\rm e}^{-{\rm i}\omega z/v}\;\nabla_{\mathbf{R}}G^{\rm ind}_{zz}(\mathbf{R},z,\mathbf{R}_0,0,\omega)\big|_{\mathbf{R}=\mathbf{R}_0}\big\},\tag{S8}$$

where ∇ has been substituted by $\nabla_{\mathbf{R}}$ and we indicate the dependence on transverse coordinates $\mathbf{R}_0$. Since the image force is conservative, we can write it in terms of a potential energy as $\mathbf{F}_{\rm im}(\mathbf{R})=-\nabla_{\mathbf{R}}V_{\rm im}(\mathbf{R})$. In particular, for our waveguide, the image potential can be found by integrating the force over the transverse coordinate x.

It is instructive to apply Eq. (S8) to a homogeneous planar surface, for which the Green tensor can be calculated analytically [8, 9]. More precisely, taking the surface at the $x=0$ plane and considering $x,x'>0$ above it, we have $G^{\rm ind}_{zz}(\mathbf{r},\mathbf{r}',\omega)=-({\rm i}/8\pi^2c^2)\int dk_y\int dk_z\,(1/k_x)\,{\rm e}^{{\rm i}[k_x(x+x')+k_y(y-y')+k_z(z-z')]}\,\big[-(k_xk_z/kk_\parallel)^2\,r_{\rm p}+(k_y/k_\parallel)^2\,r_{\rm s}\big]$, where $k_\parallel=\sqrt{k_y^2+k_z^2}$ and $k_x=\sqrt{k^2-k_\parallel^2+{\rm i}0^+}$ (with ${\rm Im}\{k_x\}>0$) are the in- and out-of-plane electromagnetic wave vectors, respectively, while $r_{\rm p}$ and $r_{\rm s}$ are the Fresnel reflection coefficients for p and s polarization. Inserting this expression in Eq. (S8), the z integral yields $\delta(k_z-\omega/v)$. After some algebra, we obtain $\mathbf{F}_{\rm im}(\mathbf{R})=F_{\rm im}(x)\,\hat{\mathbf{x}}$ with

$$F_{\rm im}(x)=-\frac{2e^2}{\pi v}\int_0^\infty d\omega\int_0^\infty dk_y\;{\rm e}^{-2\kappa x}\,\Big[(1-k^2/k_\parallel^2)\,{\rm Re}\{r_{\rm p}\}+(k_y/k_\parallel)^2\,(v/c)^2\,{\rm Re}\{r_{\rm s}\}\Big],$$

where $\kappa=\sqrt{(\omega/v\gamma)^2+k_y^2}$ and $k_\parallel=\sqrt{(\omega/v)^2+k_y^2}$. Finally, specifying this result for a homogeneous material of constant permittivity ϵ, and changing the integration variables to κ and φ with $k_y=\kappa\sin\varphi$ and $\omega/v\gamma=\kappa\cos\varphi$, we find

$$F_{\rm im}(x)=-\frac{e^2}{4x^2}\,f_{\epsilon,v},\tag{S9}$$

where

$$f_{\epsilon,v}=\frac{2\gamma}{\pi}\int_0^{\pi/2}\frac{d\varphi}{\gamma^2\cos^2\varphi+\sin^2\varphi}\,\Big[{\rm Re}\{r_{\rm p}\}+\sin^2\varphi\,(v/c)^2\,{\rm Re}\{r_{\rm s}\}\Big]$$

is a velocity- and material-dependent correction factor in which we use $r_{\rm p}=({\rm i}\epsilon-\eta)/({\rm i}\epsilon+\eta)$, $r_{\rm s}=({\rm i}-\eta)/({\rm i}+\eta)$, and $\eta=\sqrt{(\epsilon v^2/c^2-1)\gamma^2\cos^2\varphi-\sin^2\varphi+{\rm i}0^+}$ with ${\rm Re}\{\eta\}>0$.

For the rectangular waveguide considered in our work, we calculate the image force numerically from Eq. (S8) by inserting the Green tensor obtained using BEM. In Fig. S7, we compare the corresponding image potential energy with an analytical calculation from Eq. (S9). Both calculations are in excellent agreement, thus supporting the approximation $F_{\rm im}(x)\propto 1/x^2$.

S4. EFFECTIVE INTERACTION LENGTH

To obtain the integrated excitation probability for each waveguide mode n along the entire trajectory, we assume that the instantaneous probability per unit path length $dP_n(x)/dz$ can be calculated for an infinite parallel trajectory at each value of the electron–waveguide distance x. The electron interacts with the evanescent tail of the waveguide mode, whose associated field intensity exhibits an exponential decay $\propto {\rm e}^{-2x/\lambda_{\perp n}}$, also inherited by

$$\frac{dP_n(x)}{dz}\approx{\rm e}^{-2x/\lambda_{\perp n}}\,\frac{dP_n(x=0)}{dz}\tag{S10}$$

(see Fig. S8). The parallel trajectory approximation in the calculation of $dP_n(x)/dz$ should be a good approximation when the distance traveled by the electron along x is small compared to $\lambda_{\perp n}$ after propagation over a large number of mode wavelengths along z (i.e., when we have a small incidence angle relative to the waveguide).

We write the integrated probability as $P_n = \int_{-\infty}^{\infty} dz\, dP_n[x_e(z)]/dz$, where $x = x_e(z)$ defines the electron trajectory. Using the transformation $dz \to (dz/dt)(dt/dx)dx \to (v/v_x)dx$ and the fact that the trajectory is symmetric with respect to the turning point at $x = b$ (see Sec. S2), we can recast the probability into $P_n = 2v \int_b^{\infty} \left[dx/v_x(x)\right] dP_n(x)/dz$. Now, from the exponential decay of the loss probability in Eq. (S10), this expression leads to

$$P_n = L_n^{\rm eff} \times \frac{dP_n(x=0)}{dz},$$

where

$$L_n^{\rm eff} = 2v \int_b^{\infty} dx\, \frac{{\rm e}^{-2x/\lambda_{\perp n}}}{v_x(x)}$$

is the effective interaction length associated with mode n. Using Eq. (S6) for the normal velocity and changing the integration variable to $\xi = 2(x-b)/\lambda_{\perp n}$, we finally obtain

$$L_n^{\rm eff} = {\rm e}^{-2b/\lambda_{\perp n}} \sqrt{\frac{\pi m_{\rm e} \gamma v^2 \lambda_{\perp n}}{eE_{\rm DC}}} \times g_{\rm im},$$

where

$$g_{\rm im} = \frac{1}{\sqrt{\pi}} \int_0^{\infty} \frac{d\xi\, {\rm e}^{-\xi}}{\sqrt{\xi}} \left[1 + \frac{1}{1+\xi\lambda_{\perp n}/2b} \frac{F_{\rm im}(b)}{eE_{\rm DC}}\right]^{-1/2}$$

is a correction factor that accounts for the image interaction (i.e., $g_{\rm im} = 1$ if $F_{\rm im} = 0$) and produces an increase in $L_n^{\rm eff}$ because $F_{\rm im} < 0$.

References

[1] X. M. Bendaña, A. Polman, and F. J. García de Abajo, Nano Lett. **11**, 5099 (2011).
[2] V. Di Giulio, M. Kociak, and F. J. García de Abajo, Optica **6**, 1524 (2019).
[3] C. W. Johnson, A. E. Turner, and B. J. McMorran, Phys. Rev. Research **3**, 043009 (2021).
[4] C. W. Johnson, A. E. Turner, F. J. García de Abajo, and B. J. McMorran, Phys. Rev. Lett. **128**, 147401 (2022).
[5] F. J. García de Abajo and C. I. Velasco, ACS Nano **19**, 35770 (2025).
[6] D. J. Griffiths, *Introduction to Electrodynamics* (Pearson Education, Inc., Boston, 2013), 4th ed., ISBN 978-0-321-85656-2.
[7] F. J. García de Abajo, Rev. Mod. Phys. **82**, 209 (2010).
[8] S. Y. Buhmann, *Dispersion Forces I. Macroscopic Quantum Electrodynamics and Ground-State Casimir, Casimir-Polder and van der Waals Forces* (Springer-Verlag Berlin Heidelberg, Verlag Berlin Heidelberg, 2012).
[9] B. Schuler, K. A. Cochrane, C. Kastl, E. S. Barnard, E. Wong, N. J. Borys, A. M. Schwartzberg, D. F. Ogletree, F. J. García de Abajo, and A. Weber-Bargioni, Sci. Adv. **6**, eabb5988 (2020).
[10] F. J. García de Abajo and A. Howie, Phys. Rev. B **65**, 115418 (2002).
[11] E. D. Palik, *Handbook of Optical Constants of Solids* (Academic Press, San Diego, 1985).

SUPPLEMENTARY FIGURES

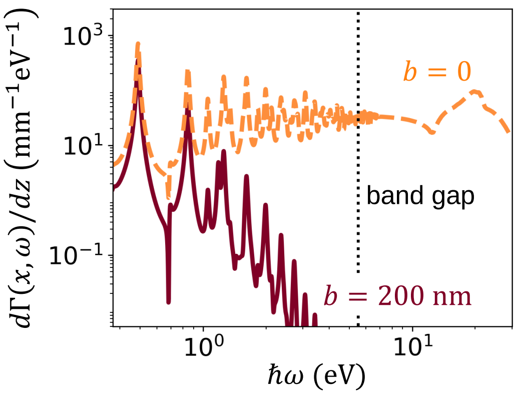

FIG. S1: **Faster decay of higher-energy inelastic losses with increasing electron–waveguide distance.** We plot the spectrally resolved loss probability per unit path length for a 200 keV electron at electron–waveguide distances $x = 0$ and $x = 200$ nm in the configuration shown in Fig. 1 of the main text (a diamond waveguide of width $W = 600$ nm and height $h = 800$ nm). Calculations are performed using BEM [10] with the frequency-dependent dielectric function of diamond taken from ref. [11]. Inelastic losses at high energy are suppressed at 200 nm, thus mitigating their detrimental effect on the electron coherence.

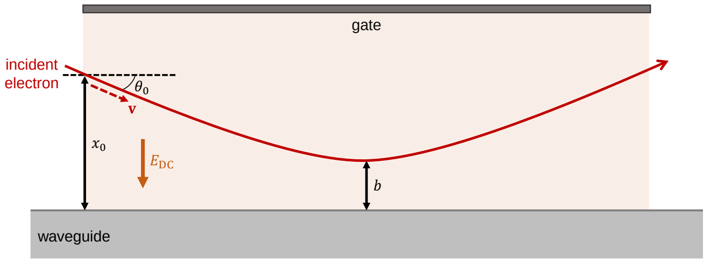

FIG. S2: **Aloof electrostatic electron bouncing from the waveguide.** An incident electron of velocity **v** enters the interaction region (shaded area) with an angle of incidence θ, at a distance x_0 from the waveguide. This region is bounded by the waveguide and a top gate, such that a potential difference applied between these two components generates a uniform electric field $E_{\rm DC}$, which repels the electron from the waveguide and produces a quasi-parabolic trajectory. The minimum electron–waveguide separation b occurs near the midpoint of the interaction region.

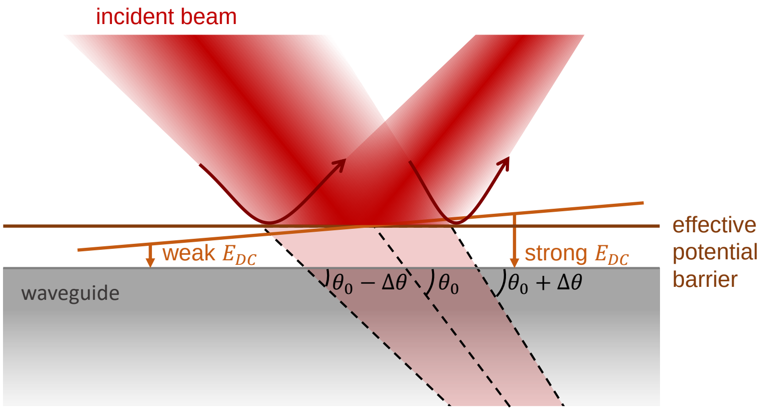

FIG. S3: **Uniform deflection of a divergent beam.** A wide electron beam having an angular divergence $\Delta\theta$ is directed toward the waveguide, centered around an incidence angle θ_0. Before reaching the waveguide surface, the beam is deflected by a DC electric field $E_{\rm DC}$. We propose to increase this field along the waveguide surface in a way such that e-beam components with larger incidence angles experience a stronger repelling field, thus compensating for their larger normal kinetic energy. With a proper adjustment of the field increase along the waveguide surface, all angular e-beam components can be reflected at a similar distance from the waveguide.

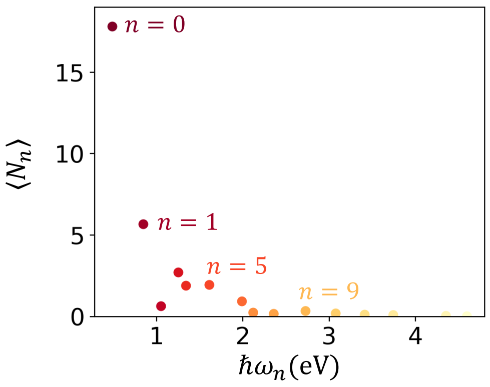

FIG. S4: **Photon generation probability under the conditions of Fig. 4c in the main text.** We plot the average number of photons generated in different modes n by a 200 keV electron in the coupling configuration of Fig. 1a for a diamond waveguide of width $W = 600$ nm and height $h = 800$ nm. The amplitude of the perpendicular DC field is $E_{\rm DC} = 25$ V/mm and the minimum electron–waveguide distance is $b = 200$ nm. The horizontal axis indicates the mode frequency.

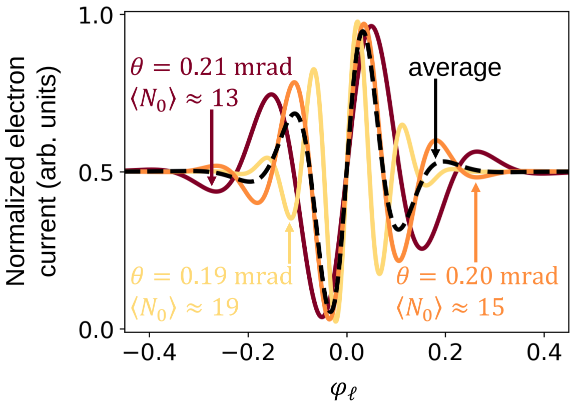

FIG. S5: **Tolerance of QUAFE to angular divergence of the incident e-beam.** Under the conditions of Fig. 4c in the main text, we calculate the current obtained for different electron incidence angles θ (solid curves, see labels and the corresponding average numbers of generated waveguided photons), along with the average of these curves over a Gaussian distribution $G(\theta) = \exp\big[-(\theta - \theta_0)^2/(2\Delta\theta^2)\big]$ of width $\Delta\theta = 0.01$ mrad centered around $\theta_0 = 0.2$ mrad ($\gg \Delta\theta$). For each angle θ, a different transmitted current $I(\theta)$ is produced, and the average current (dashed curve) is obtained from $\int d\theta\, G(\theta) I(\theta)$. The waveguide parameters are the same as in Fig. 1 of the main text. We set the electron energy to $\mathcal{E}_0 = 200$ keV and the minimum electron–waveguide distance to $b = 200$ nm for the central e-beam component at $\theta = \theta_0$.

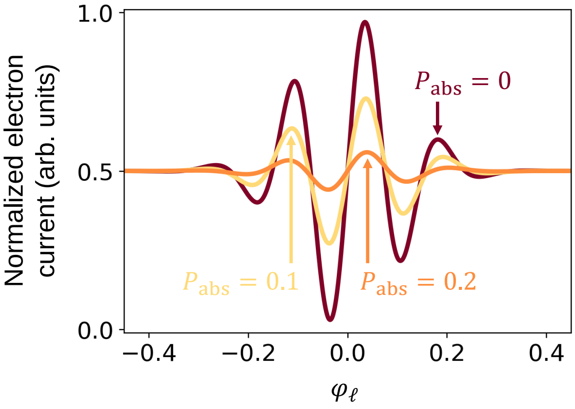

FIG. S6: **Tolerance of QUAFE to photon loss between the two electron–waveguide interaction points**. Under the conditions in Fig. 4 of the main text for an incidence angle $\theta_0 = 0.2$ mrad, we calculate the current obtained when introducing inelastic losses in the optical waveguide through a photon absorption probability $P_{\rm abs}$ during light propagation from point 1 to point 2 in the waveguide (see Fig. 4a,b in the main text). Each curve corresponds to a different absorption probability. The waveguide parameters are the same as in Fig. 1 of the main text for an electron energy set to $\mathcal{E}_0 = 200$ keV.

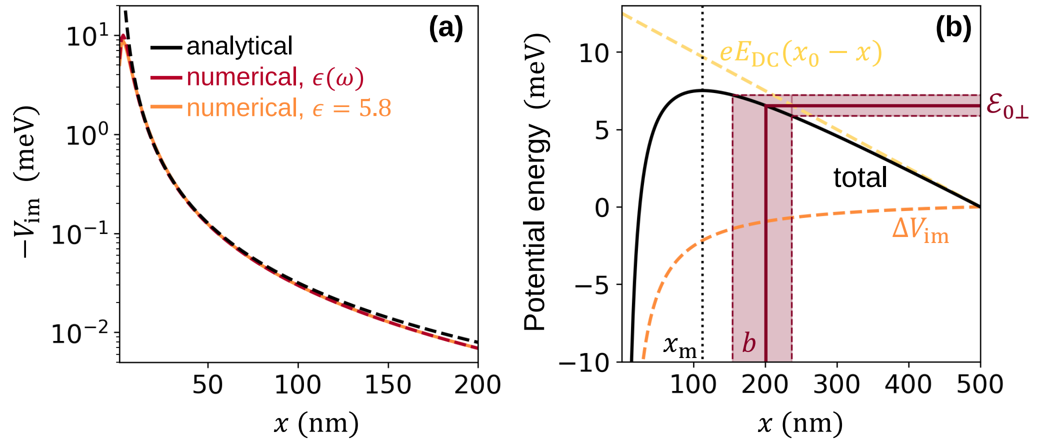

FIG. S7: **Effect of the image potential on the electron trajectory**. **(a)** Image potential energy $V_{\rm im}(x)$ acting on a 200 keV electron traveling parallel to the diamond waveguide considered in Fig. 1 of the main text as a function of distance x from a waveguide surface. We compare the results obtained by assuming different approximations to compute the image potential. Numerical calculations for $V_{\rm im}(x)$ rely on BEM with the frequency-dependent dielectric function of diamond taken from ref. [11] (solid-red curve) or set to a constant value $\epsilon = 5.8$ (solid-orange curve). For comparison, we also plot the analytical expression $-e^2 f_{\epsilon,v}/4x$ (dashed curve), describing the image potential energy for an electron moving at a distance x from a semi-infinite medium of permittivity $\epsilon = 5.8$. Here, $f_{\epsilon,v}$ is a correction factor that depends on ϵ and electron velocity v. **(b)** Potential energy difference as a function of x measured relative to $x_0 = 500$ nm. The total potential energy (solid curve) is the sum of the linear contribution produced by the DC field $E_{\rm DC}$ (dashed-yellow line) and the image potential energy (dashed-orange curve). The horizontal solid line indicates the transverse kinetic energy $\mathcal{E}_{0\perp} \approx m_{\rm e}\gamma v^2\theta_0^2/2$ for a 200 keV electron incident with an angle $\theta_0 = 0.2$ mrad. The intersection between $\mathcal{E}_{0\perp}$ and the total potential energy defines the minimum electron–waveguide distance b (200 nm in this case). The shaded region around $\mathcal{E}_{0\perp}$ represents the uncertainty in transverse energy due to an angular divergence in the e-beam given by $\pm\Delta\theta = 0.01$ mrad, which results in an uncertainty in b. The total potential reaches a maximum at $x_{\rm m}$, where the attractive image force cancels the repulsive force from the DC field.

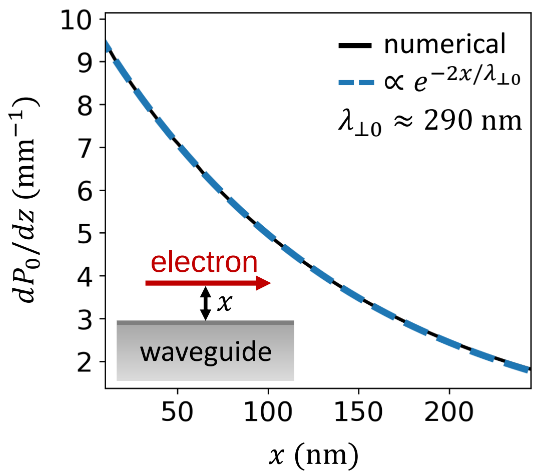

FIG. S8: **Exponential mode decay in vacuum outside the waveguide.** Dependence of the photon-generation probability $dP_0(x)/dz$ of mode $n = 0$ on electron–waveguide distance x calculated from BEM (solid curve) and exponential fit $\propto e^{-2x/\lambda_{\perp 0}}$ for a decay length $\lambda_{\perp 0} \approx 290$ nm (dashed curve).
